# Treatment of prosthetic vascular graft infection in the groin with ultrasound debridement: A case report

**DOI:** 10.1016/j.amsu.2020.10.037

**Published:** 2020-10-21

**Authors:** Makoto Haga, Hidenori Inoue, Shunya Shindo

**Affiliations:** Department of Cardiovascular Surgery, Tokyo Medical University Hachioji Medical Center, Tokyo, Japan

**Keywords:** Chronic limb-threatening ischemia, Prosthetic graft infection, Ultrasound debridement, Case report

## Abstract

**Introduction:**

Prosthetic graft infection (PGI) is associated with low patient survival rates. The effectiveness of ultrasound debridement in chronic wound healing has been previously reported; however, data on the use of ultrasound technology and its effect on the treatment of PGI are still lacking. We report a case in which PGI in the groin was managed by graft removal using ultrasound debridement.

**Presentation of case:**

A 70-year-old man was diagnosed with chronic limb-threatening ischemia and underwent a femoral-femoral bypass with a polytetrafluoroethylene graft. Eight months postoperatively, he developed an infection at the femoral incision site. Graft removal was performed using ultrasound debridement. The estimated blood loss was 10 mL. The wound healed, and the patient has remained in good health for 2 years postoperatively.

**Discussion:**

When the ultrasonic probe is applied to the wound, ultrasonic energy penetrates into the tissue, and a fibrinolytic action removes necrotic or infected tissue without removing healthy tissue, thereby minimizing bleeding. Using this technique, we were able to perform effective debridement at not only the wound but also the anastomosis.

**Conclusion:**

It is our opinion that this technique can be used to achieve adequate debridement with little bleeding during graft removal and may provide a new option for the treatment of PGI.

## Highlights

• Prosthetic graft infection (PGI) is associated with low patient survival rates.
• We present a case in which PGI in the groin was managed by graft removal using ultrasound debridement (UD).
• Ultrasonic energy penetrates into the tissue and cleaves dead tissue without removing healthy tissue.
• UD can be used to achieve adequate debridement during graft removal and may provide a new option for the treatment of PGI.

## 1 Introduction

Prosthetic graft infection (PGI) is associated with low patient survival rates and poor limb salvage in patients with peripheral arterial disease. Traditionally, the treatment of PGI has included removing the infected prosthesis, administering antibiotics, and performing an extra-anatomic bypass. The effectiveness of ultrasound debridement in chronic wound healing has been previously reported [1]. However, evidence supporting the use of ultrasound technology and describing its effect on the treatment of PGI is still lacking. Here, we report a case in which PGI in the groin was managed by graft removal using ultrasound debridement.

## 2 Methods

**Fig. 1:**
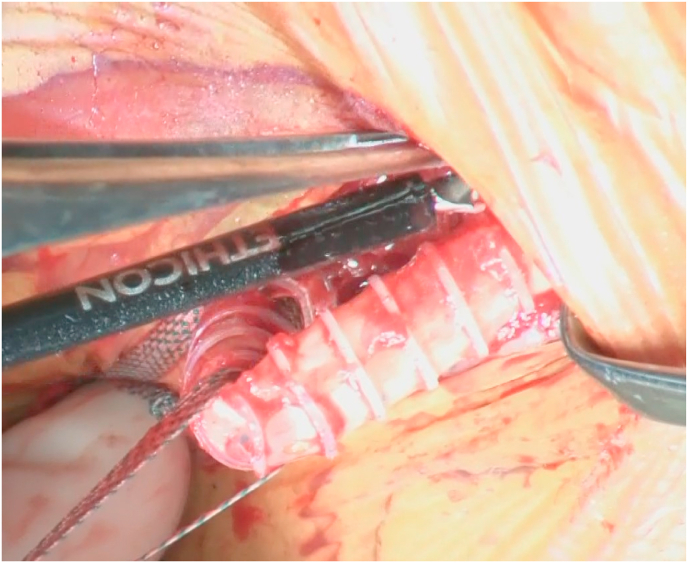
Graft removal and debridement performed with a harmonic scalpel.

The graft removal and debridement were performed with an ultrasonically activated scalpel (Harmonic Scalpel 5-mm Dissecting Hook; Ethicon Endo-Surgery, USA, Fig. 1). The probe was activated by an ultrasonic generator, and the probe of the ultrasound device vibrates at 26,000–55,500 Hz with a vibratory amplitude of 15–100 μm per stroke. We chose the hook blade and not the curved or ball blade because we were able to determine whether the tissue was necrotized by hooking and grasping it with the hook blade. This work was reported in line with the SCARE 2018 criteria [2].

## 3 Presentation of Case

**Fig. 2:**
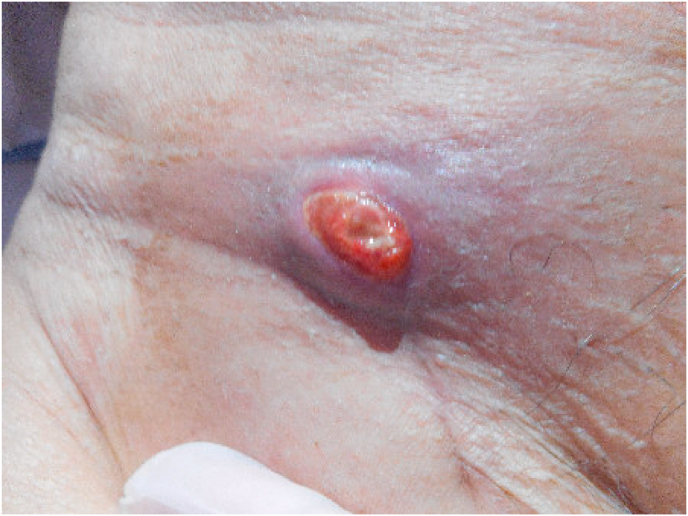
Wound infection in the right groin of the patient.

**Fig. 3:**
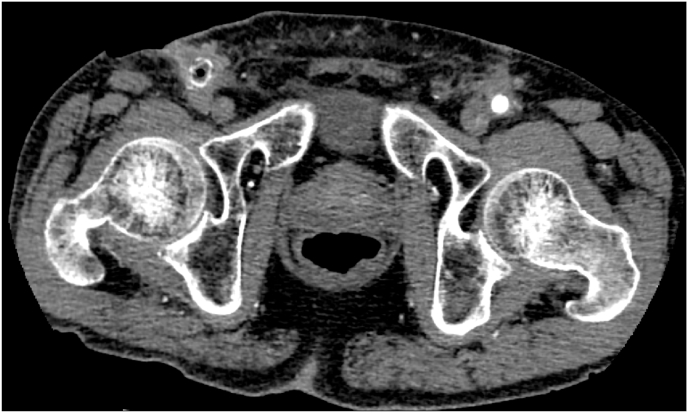

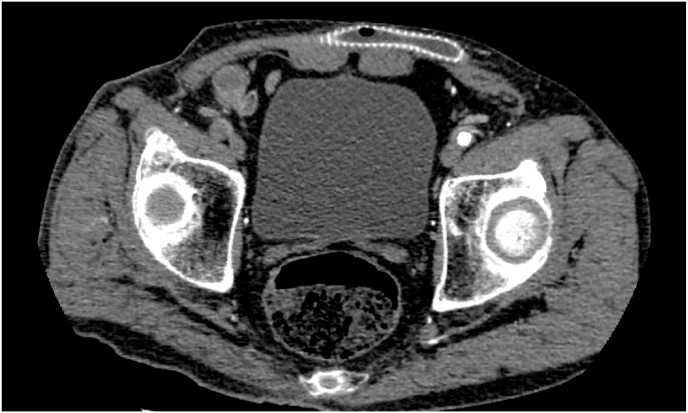
A) Computed tomography showing an infected abscess in contact with an air bubble in the graft. B) Computed tomography showing an air bubble in the femoral-femoral crossover bypass graft.

A 70-year-old man presented to our hospital with rest pain and severe claudication in the right leg. He was diagnosed with chronic limb-threatening ischemia with an occluded right common iliac artery using computed tomography (CT). He initially underwent an endovascular revascularization a few years prior in a different hospital, but it failed. Therefore, we underwent a femoral-femoral crossover bypass with a polytetrafluoroethylene graft. He recovered well from this procedure and was discharged to return home 10 days postoperatively. Duplex scans were performed at follow-ups every 3 months, which indicated no significant problems. However, 8 months after the crossover surgery, he presented to our hospital with claudication in the right leg and an infected abscess in contact with the graft at the right femoral incision site (Fig. 2). CT revealed an air bubble and total occlusion in the prosthetic graft, as well as an infection that surrounded the anastomosis and involved the body of the graft (Fig. 3A). An air bubble was also identified in the middle of the prosthetic graft (Fig. 3B). The common femoral arteries were both patent bilaterally. The vascular graft infection classification was group 4 (Samson Classification). The patient was readmitted, and subsequent bacterial culture confirmed infection of the graft with aerobic bacteria (*Staphylococcus lugdunensis* and *Corynebacterium*). Treatment with ceftriaxone, vancomycin, and local drainage was initiated. Despite continuous antibiotic treatment, CT showed that an air bubble remained 1 week later. Therefore, we performed a graft removal.

**Fig. 4:**
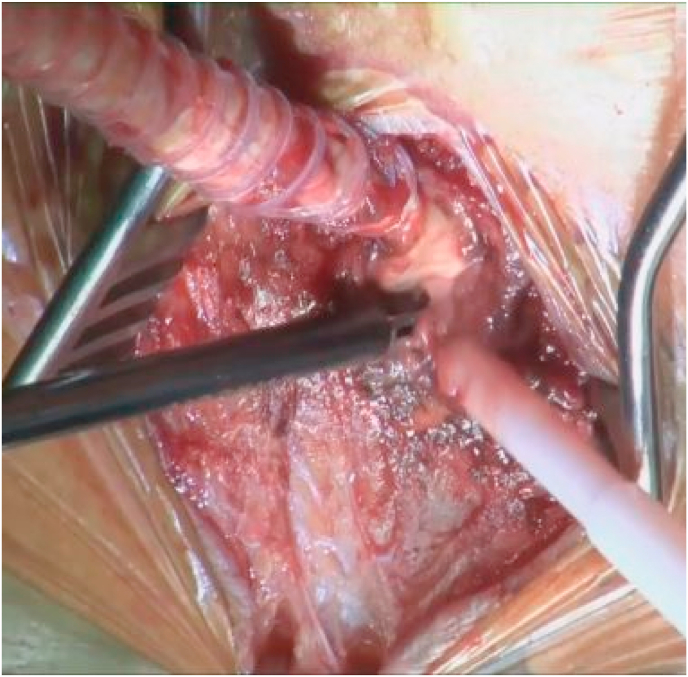
Ultrasound debridement performed at the right femoral anastomosis.

MH and HI performed the procedure (operator experience including more than 10 years of specialized training). A bilateral groin and midline incision was made while the patient was under general anesthesia and in the supine position. The infected prosthetic graft was occluded by thrombosis, and it was extremely incorporated with surrounding tissues; therefore, extensive manipulation and debridement were required to remove the graft. The prosthetic graft was partially removed by ligating 3 cm from the left femoral anastomosis without disrupting the anastomosis because we did not find any significant infection in that region. The graft was removed completely from the right side of the groin, and a midline incision was made with an ultrasonically activated scalpel. The surrounding tissues received focused treatment (with the hook moving over the surface) until the infected areas and fibrin were removed; notably, the tissue appeared macroscopically normal upon the surgeon's inspection. The wall of the right common femoral artery was debrided ultrasonically (Fig. 4) and sutured with 5–0 polypropylene sutures. All the incisions were closed without the need to insert a surgical drain. The operation was completed in 2 hours and 28 minutes. The estimated blood loss was 10 mL.

The postoperative period of the patient was uneventful. The patient continued antibiotics until postoperative day 7 and was discharged on postoperative day 11. The wound was fully healed 1 month later. Claudication remained in the right leg; however, as of 2 years postoperatively, no significant problems have occurred.

## 4 Discussion

Peripheral graft infections occur in approximately 4% of cases and may lead to limb loss [3,4]. When infection occurs, the resulting morbidity and mortality rates are high [3,5]. Therefore, aggressive treatment is typically used: graft removal with debridement, followed by ex situ bypass and antibiotic therapy. However, there are no clear guidelines for the management of PGI. Some authors advocate performing a less-invasive surgery, with a more conservative approach. Conversely, other surgeons advocate aggressive treatment, including complete graft removal with extensive debridement. Our basic strategy for PGI is complete removal, and in the present report, we used ultrasound to treat the PGI and perform debridement in the groin.

Ultrasound treatment for cutting and coagulating tissues was originally developed for use in abdominal surgery, particularly with regard to laparoscopy [6,7]. Currently, ultrasound treatment is often used to promote wound healing, and its advantages have been established [1,8]. The effects of applying ultrasonic waves to biologic tissue are primarily due to cavitation and acoustic streaming [9]. Notably, cavitation is a vibratory energy that can be applied to remove necrotic material from the wound bed. When ultrasonic energy comes into contact with tissue, microbubbles form at nucleation sites [10]. The microbubbles oscillate under pressure and will collapse when exposed to energy of sufficient magnitude. Importantly, the collapse of these microbubbles (both in tissue fluids and in the external liquid used for acoustic coupling) provides the stimulus for mechanical debridement; then, when the probe is applied to the wound, ultrasonic energy penetrates into the tissue, and the fibrinolytic action removes necrotic or infected tissue without removing healthy tissue. Therefore, during graft removal, it is possible to achieve adequate debridement with less bleeding than with the use of an electric scalpel.

Applying this ultrasound debridement technique on the anastomosis is the most important and effective component of this procedure. The main bleeding point during the removal of an infected graft is usually at or near the anastomosis because collateral vessels often appear near the anastomosis and because the walls of the vessels are fragile because of the infection. Ultrasonic energy can be used to induce coagulation in small collateral vessels and remove the infected tissue without disrupting healthy tissue, thereby minimizing bleeding. In addition, because, in our opinion, the ultrasound debridement completely removed the infected tissue, we were able to simply close the wound without inserting a surgical drain or performing negative pressure wound therapy.

Conversely, the main disadvantage of this technique is the high cost of using this equipment. The cost of using an ultrasonically activated scalpel is approximately $900. The Harmonic Scalpel is relatively expensive, and there are additional costs for disposable single-use shears, cleaning of the shears, and sterilization of the shears and hand piece. However, this procedure can be performed on an outpatient basis and may reduce direct staff costs [1]. Without the use of this equipment, the condition of many of these patients may otherwise develop into a critical situation, requiring a more expensive and invasive surgical treatment.

A second disadvantage of ultrasound debridement is that the prolonged debridement of the wall of the graft can impact the impermeability of the graft or cause considerable damage to the graft. Thus, the safety and effectiveness of debridement or of the removal of vein grafts using this procedure are unknown. Careful manipulation of the probe is recommended during the procedure.

Because this report involved a single case, no definite conclusions can be made regarding the suitability of the approach for other patients with PGI. A larger series of patients, including an analysis of the functional long-term outcomes of affected patients, is needed to confirm the findings of the present case.

## 5Conclusion

We described a case in which PGI in the groin was managed by ultrasound debridement with graft removal. It is our opinion that this technique can be used to achieve adequate debridement with little bleeding during graft removal and may provide a new option for the treatment of PGI.

## Provenance and peer review

Not commissioned, externally peer-reviewed.

## Annals of medicine and surgery

The following information is required for submission. Please note that failure to respond to these questions/statements will mean your submission will be returned. If you have nothing to declare in any of these categories then this should be stated.

## Registration of research studies

In accordance with the Declaration of Helsinki 2013, all research involving human participants has to be registered in a publicly accessible database. Please enter the name of the registry and the unique identifying number (UIN) of your study.

You can register any type of research at http://www.researchregistry.com to obtain your UIN if you have not already registered. This is mandatory for human studies only. Trials and certain observational research can also be registered elsewhere such as: ClinicalTrials.gov or ISRCTN or numerous other registries.

Not applicable.
